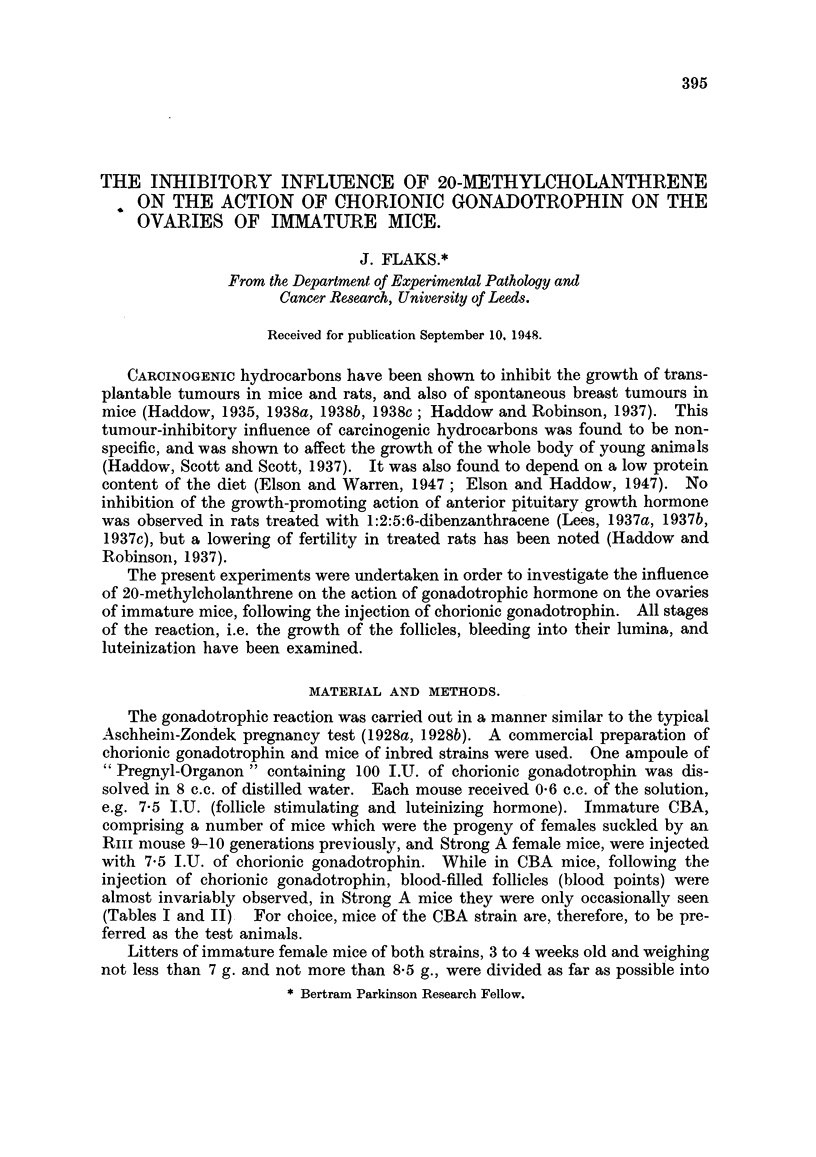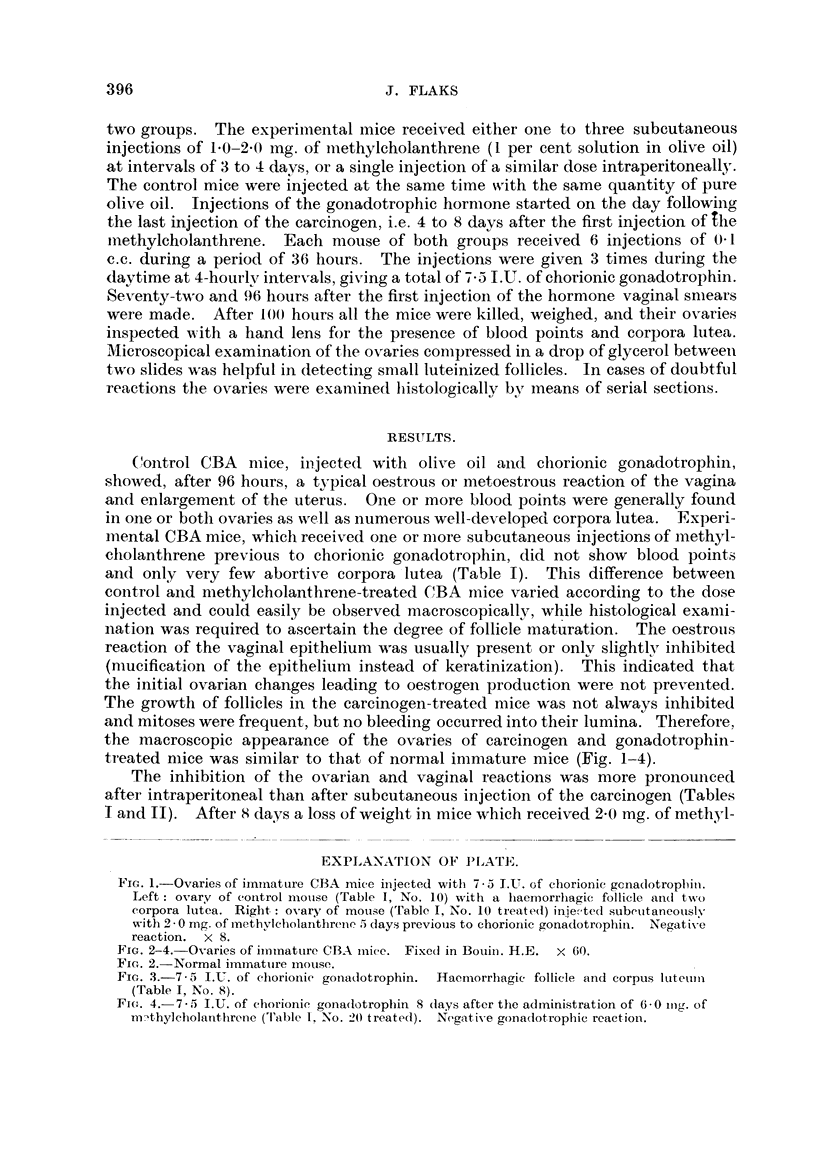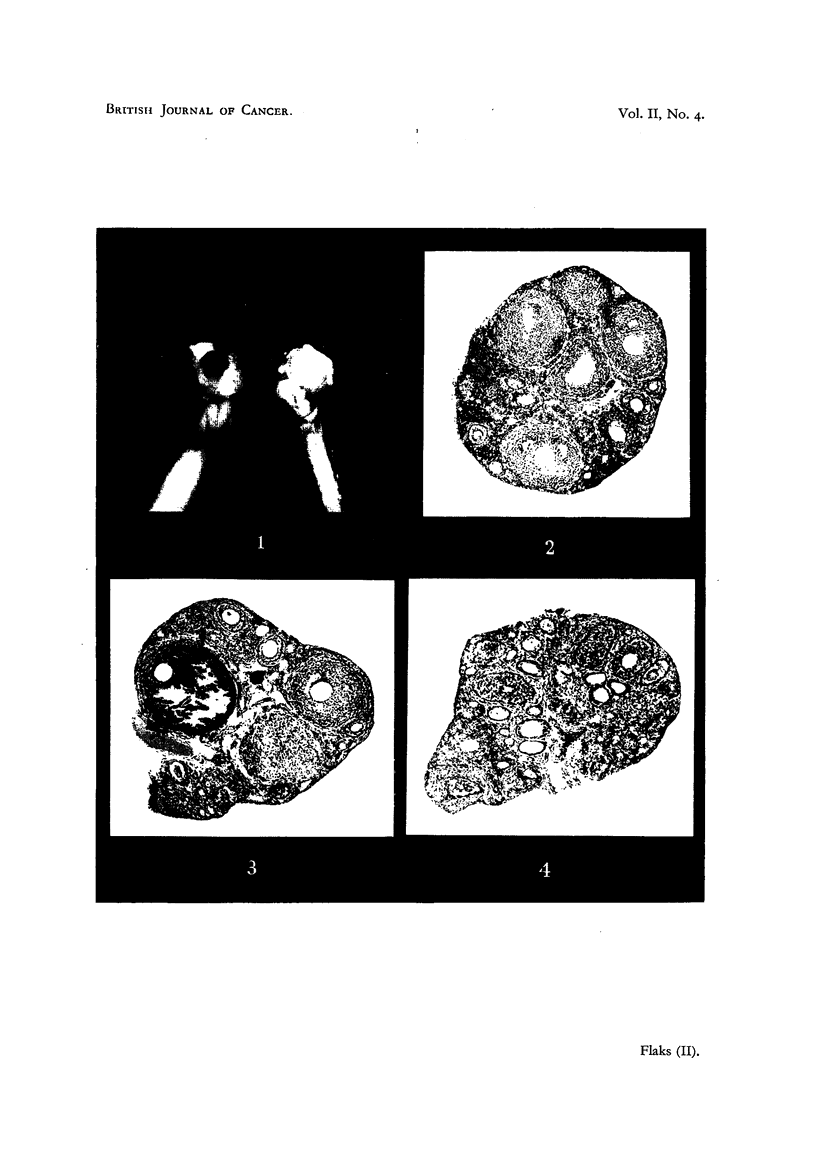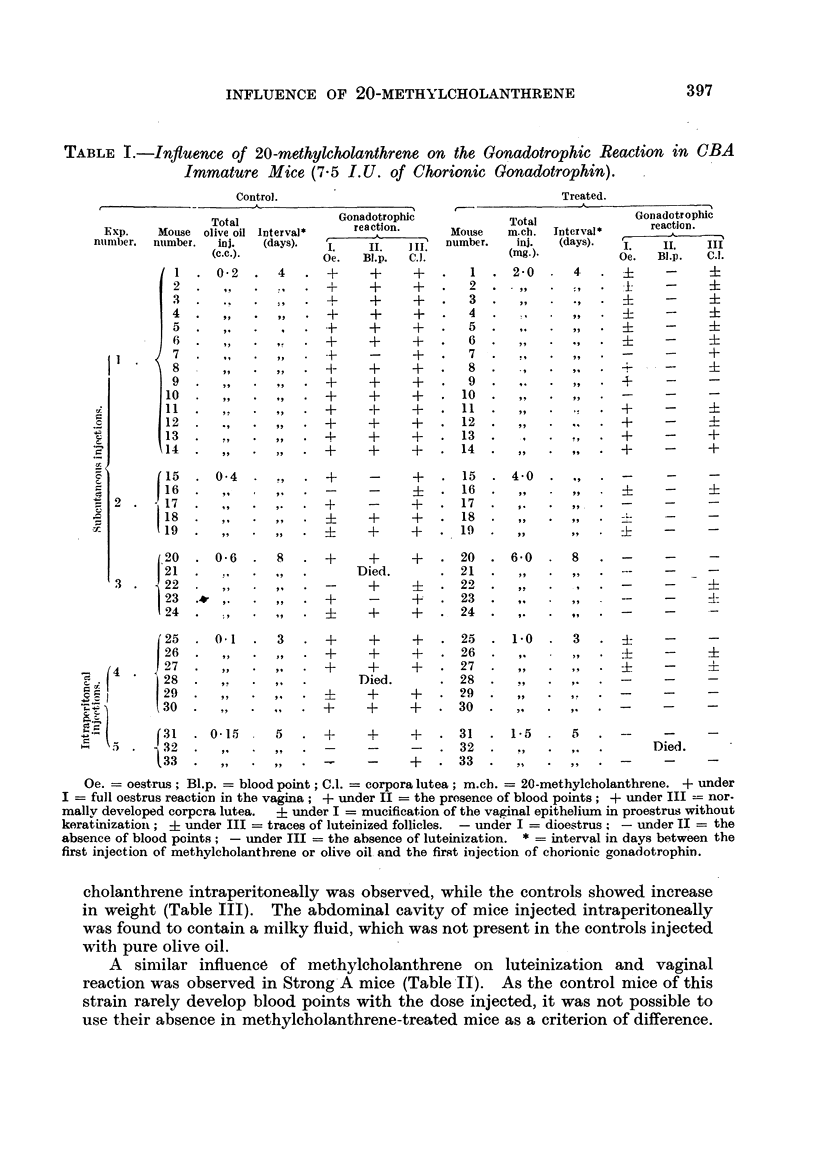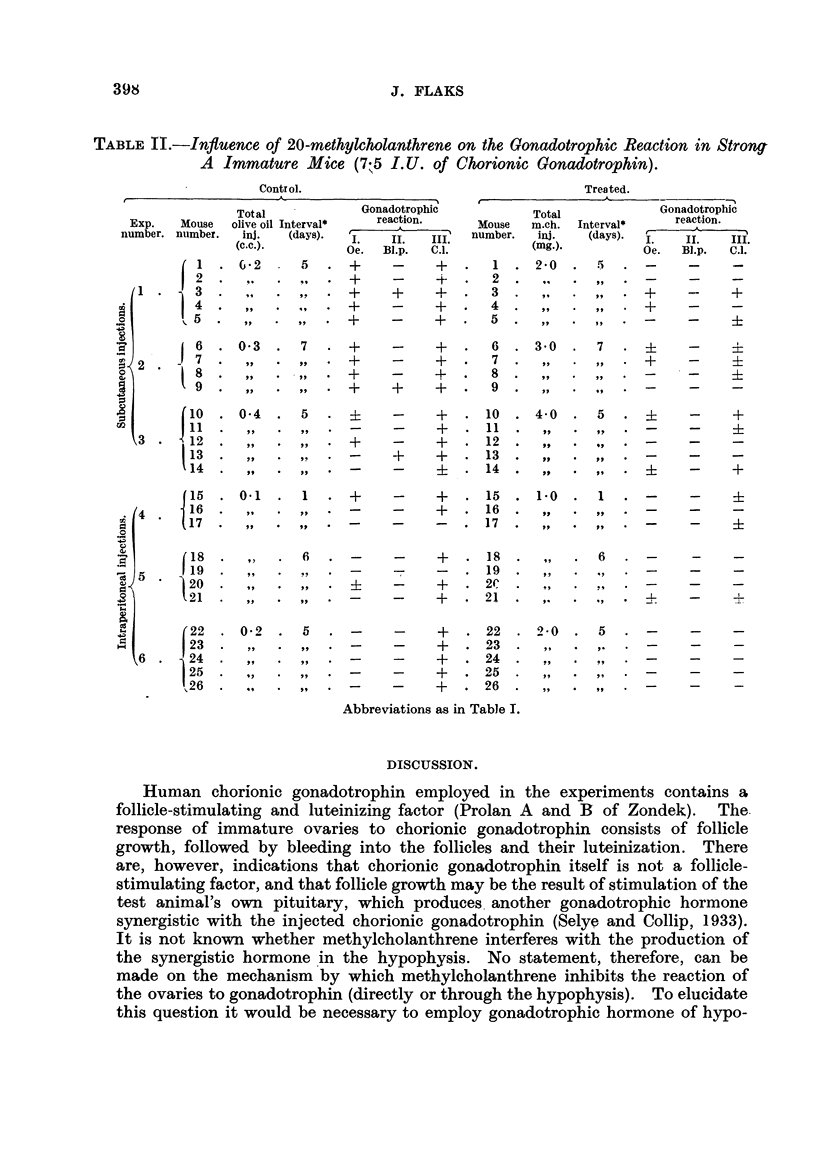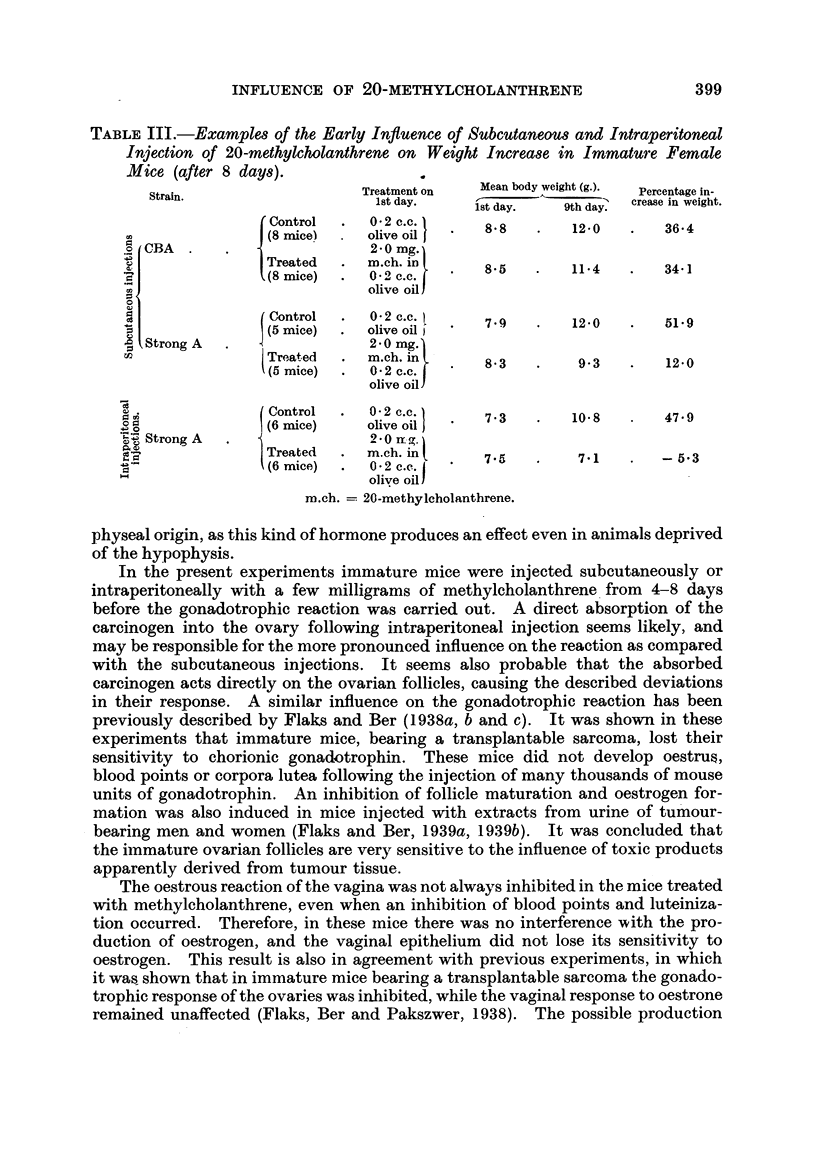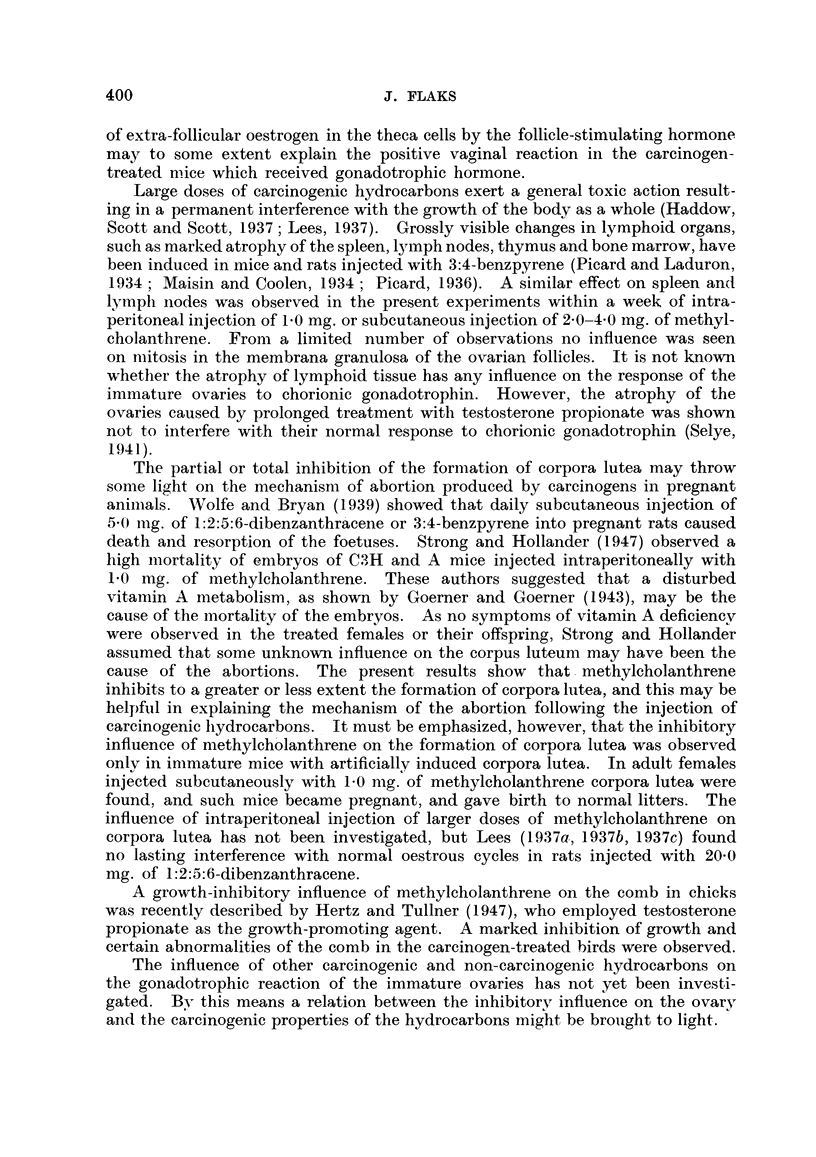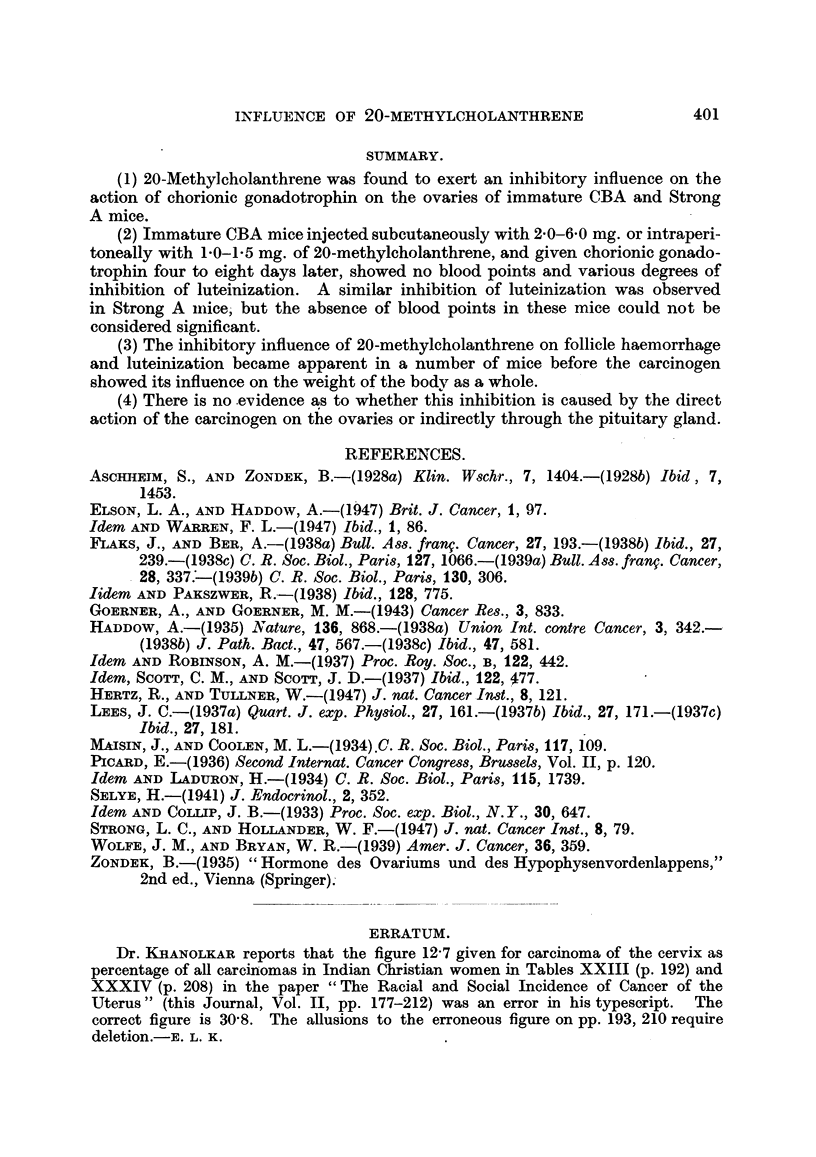# The Inhibitory Influence of 20-Methylcholanthrene on the Action of Chorionic Gonadotrophin on the Ovaries of Immature Mice

**DOI:** 10.1038/bjc.1948.44

**Published:** 1948-12

**Authors:** J. Flaks

## Abstract

**Images:**


### THE INHIBITORY INFLUENCE OF 20-METHYLCHOLANTHRENE ON THE ACTION OF CHORIONIC GONADOTROPHIN ON THE OVARIES OF IMMATURE MICE.

J. FLAKS.*

*From the Department of Experimental Pathology and Cancer Research, University of Leeds.*

Received for publication September 10, 1948.

CARCINOGENIC hydrocarbons have been shown to inhibit the growth of transplantable tumours in mice and rats, and also of spontaneous breast tumours in mice (Haddow, 1935, 1938*a*, 1938*b*, 1938*c*; Haddow and Robinson, 1937). This tumour-inhibitory influence of carcinogenic hydrocarbons was found to be non-specific, and was shown to affect the growth of the whole body of young animals (Haddow, Scott and Scott, 1937). It was also found to depend on a low protein content of the diet (Elson and Warren, 1947; Elson and Haddow, 1947). No inhibition of the growth-promoting action of anterior pituitary growth hormone was observed in rats treated with 1:2:5:6-dibenzanthracene (Lees, 1937*a*, 1937*b*, 1937*c*), but a lowering of fertility in treated rats has been noted (Haddow and Robinson, 1937).

The present experiments were undertaken in order to investigate the influence of 20-methylcholanthrene on the action of gonadotrophic hormone on the ovaries of immature mice, following the injection of chorionic gonadotrophin. All stages of the reaction, i.e. the growth of the follicles, bleeding into their lumina, and luteinization have been examined.

### MATERIAL AND METHODS.

The gonadotrophic reaction was carried out in a manner similar to the typical Aschheim-Zondek pregnancy test (1928*a*, 1928*b*). A commercial preparation of chorionic gonadotrophin and mice of inbred strains were used. One ampoule of "Pregnyl-Organon" containing 100 I.U. of chorionic gonadotrophin was dissolved in 8 c.c. of distilled water. Each mouse received 0·6 c.c. of the solution, e.g. 7·5 I.U. (follicle stimulating and luteinizing hormone). Immature CBA, comprising a number of mice which were the progeny of females suckled by an RIII mouse 9–10 generations previously, and Strong A female mice, were injected with 7·5 I.U. of chorionic gonadotrophin. While in CBA mice, following the injection of chorionic gonadotrophin, blood-filled follicles (blood points) were almost invariably observed, in Strong A mice they were only occasionally seen (Tables I and II). For choice, mice of the CBA strain are, therefore, to be preferred as the test animals.

Litters of immature female mice of both strains, 3 to 4 weeks old and weighing not less than 7 g. and not more than 8·5 g., were divided as far as possible into

* Bertram Parkinson Research Fellow.

two groups. The experimental mice received either one to three subcutaneous injections of 1·0–2·0 mg. of methylcholanthrene (1 per cent solution in olive oil) at intervals of 3 to 4 days, or a single injection of a similar dose intraperitoneally. The control mice were injected at the same time with the same quantity of pure olive oil. Injections of the gonadotrophic hormone started on the day following the last injection of the carcinogen, i.e. 4 to 8 days after the first injection of the methylcholanthrene. Each mouse of both groups received 6 injections of 0·1 c.c. during a period of 36 hours. The injections were given 3 times during the daytime at 4-hourly intervals, giving a total of 7·5 I.U. of chorionic gonadotrophin. Seventy-two and 96 hours after the first injection of the hormone vaginal smears were made. After 100 hours all the mice were killed, weighed, and their ovaries inspected with a hand lens for the presence of blood points and corpora lutea. Microscopical examination of the ovaries compressed in a drop of glycerol between two slides was helpful in detecting small luteinized follicles. In cases of doubtful reactions the ovaries were examined histologically by means of serial sections.

### RESULTS.

Control CBA mice, injected with olive oil and chorionic gonadotrophin, showed, after 96 hours, a typical oestrous or metoestrous reaction of the vagina and enlargement of the uterus. One or more blood points were generally found in one or both ovaries as well as numerous well-developed corpora lutea. Experimental CBA mice, which received one or more subcutaneous injections of methylcholanthrene previous to chorionic gonadotrophin, did not show blood points and only very few abortive corpora lutea (Table I). This difference between control and methylcholanthrene-treated CBA mice varied according to the dose injected and could easily be observed macroscopically, while histological examination was required to ascertain the degree of follicle maturation. The oestrous reaction of the vaginal epithelium was usually present or only slightly inhibited (mucification of the epithelium instead of keratinization). This indicated that the initial ovarian changes leading to oestrogen production were not prevented. The growth of follicles in the carcinogen-treated mice was not always inhibited and mitoses were frequent, but no bleeding occurred into their lumina. Therefore, the macroscopic appearance of the ovaries of carcinogen and gonadotrophin-treated mice was similar to that of normal immature mice (Fig. 1–4).

The inhibition of the ovarian and vaginal reactions was more pronounced after intraperitoneal than after subcutaneous injection of the carcinogen (Tables I and II). After 8 days a loss of weight in mice which received 2·0 mg. of methyl-

---

EXPLANATION OF PLATE.

Fig. 1.—Ovaries of immature CBA mice injected with 7·5 I.U. of chorionic gonadotrophin. Left: ovary of control mouse (Table I, No. 10) with a haemorrhagic follicle and two corpora lutea. Right: ovary of mouse (Table I, No. 10 treated) injected subcutaneously with 2·0 mg. of methylcholanthrene 5 days previous to chorionic gonadotrophin. Negative reaction. × 8.

Fig. 2–4.—Ovaries of immature CBA mice. Fixed in Bouin. H.E. × 60.

Fig. 2.—Normal immature mouse.

Fig. 3.—7·5 I.U. of chorionic gonadotrophin. Haemorrhagic follicle and corpus luteum (Table I, No. 8).

Fig. 4.—7·5 I.U. of chorionic gonadotrophin 8 days after the administration of 6·0 mg. of methylcholanthrene (Table I, No. 20 treated). Negative gonadotrophic reaction.

1
2
3
4

TABLE I.—*Influence of 20-methylcholanthrene on the Gonadotrophic Reaction in CBA Immature Mice (7·5 I.U. of Chorionic Gonadotrophin).*

| | | Control. | | | | | | Treated. | | | | | |
|---|---|---|---|---|---|---|---|---|---|---|---|---|---|
| | Exp. number. | Mouse number. | Total olive oil inj. (c.c.). | Interval* (days). | Gonadotrophic reaction. I. Oe. | II. Bl.p. | III. C.l. | Mouse number. | Total m.ch. inj. (mg.). | Interval* (days). | Gonadotrophic reaction. I. Oe. | II. Bl.p. | III C.l. |
| Subcutaneous injections. | 1 | 1 | 0·2 | 4 | + | + | + | 1 | 2·0 | 4 | ± | — | ± |
| | | 2 | ,, | ,, | + | + | + | 2 | ,, | ,, | ± | — | ± |
| | | 3 | ,, | ,, | + | + | + | 3 | ,, | ,, | ± | — | ± |
| | | 4 | ,, | ,, | + | + | + | 4 | ,, | ,, | ± | — | ± |
| | | 5 | ,, | ,, | + | + | + | 5 | ,, | ,, | ± | — | ± |
| | | 6 | ,, | ,, | + | + | + | 6 | ,, | ,, | ± | — | ± |
| | | 7 | ,, | ,, | + | — | + | 7 | ,, | ,, | — | — | + |
| | | 8 | ,, | ,, | + | + | + | 8 | ,, | ,, | + | — | ± |
| | | 9 | ,, | ,, | + | + | + | 9 | ,, | ,, | + | — | — |
| | | 10 | ,, | ,, | + | + | + | 10 | ,, | ,, | — | — | — |
| | | 11 | ,, | ,, | + | + | + | 11 | ,, | ,, | + | — | ± |
| | | 12 | ,, | ,, | + | + | + | 12 | ,, | ,, | + | — | ± |
| | | 13 | ,, | ,, | + | + | + | 13 | ,, | ,, | + | — | + |
| | | 14 | ,, | ,, | + | + | + | 14 | ,, | ,, | + | — | + |
| | 2 | 15 | 0·4 | ,, | + | — | + | 15 | 4·0 | ,, | — | — | — |
| | | 16 | ,, | ,, | — | — | ± | 16 | ,, | ,, | ± | — | ± |
| | | 17 | ,, | ,, | + | — | + | 17 | ,, | ,, | — | — | — |
| | | 18 | ,, | ,, | ± | + | + | 18 | ,, | ,, | ± | — | — |
| | | 19 | ,, | ,, | ± | + | + | 19 | ,, | ,, | ± | — | — |
| | 3 | 20 | 0·6 | 8 | + | + | + | 20 | 6·0 | 8 | — | — | — |
| | | 21 | ,, | ,, | | Died. | | 21 | ,, | ,, | — | — | — |
| | | 22 | ,, | ,, | — | + | ± | 22 | ,, | ,, | — | — | ± |
| | | 23 | ,, | ,, | + | — | + | 23 | ,, | ,, | — | — | ± |
| | | 24 | ,, | ,, | ± | + | + | 24 | ,, | ,, | — | — | — |
| Intraperitoneal injections. | 4 | 25 | 0·1 | 3 | + | + | + | 25 | 1·0 | 3 | ± | — | — |
| | | 26 | ,, | ,, | + | + | + | 26 | ,, | ,, | ± | — | ± |
| | | 27 | ,, | ,, | + | + | + | 27 | ,, | ,, | ± | — | ± |
| | | 28 | ,, | ,, | | Died. | | 28 | ,, | ,, | — | — | — |
| | | 29 | ,, | ,, | ± | + | + | 29 | ,, | ,, | — | — | — |
| | | 30 | ,, | ,, | + | + | + | 30 | ,, | ,, | — | — | — |
| | 5 | 31 | 0·15 | 5 | + | + | + | 31 | 1·5 | 5 | — | — | — |
| | | 32 | ,, | ,, | — | — | — | 32 | ,, | ,, | | Died. | |
| | | 33 | ,, | ,, | — | — | + | 33 | ,, | ,, | — | — | — |

Oe. = oestrus ; Bl.p. = blood point ; C.l. = corpora lutea ; m.ch. = 20-methylcholanthrene. + under I = full oestrus reaction in the vagina ; + under II = the presence of blood points ; + under III = normally developed corpora lutea. ± under I = mucification of the vaginal epithelium in proestrus without keratinization ; ± under III = traces of luteinized follicles. — under I = dioestrus ; — under II = the absence of blood points ; — under III = the absence of luteinization. * = interval in days between the first injection of methylcholanthrene or olive oil and the first injection of chorionic gonadotrophin.

cholanthrene intraperitoneally was observed, while the controls showed increase in weight (Table III). The abdominal cavity of mice injected intraperitoneally was found to contain a milky fluid, which was not present in the controls injected with pure olive oil.

A similar influence of methylcholanthrene on luteinization and vaginal reaction was observed in Strong A mice (Table II). As the control mice of this strain rarely develop blood points with the dose injected, it was not possible to use their absence in methylcholanthrene-treated mice as a criterion of difference.

TABLE II.—*Influence of 20-methylcholanthrene on the Gonadotrophic Reaction in Strong A Immature Mice (7·5 I.U. of Chorionic Gonadotrophin).*

| Route | Exp. number. | Control: Mouse number. | Control: Total olive oil inj. (c.c.). | Control: Interval* (days). | Control: Gonadotrophic reaction. I. Oe. | Control: II. Bl.p. | Control: III. C.l. | Treated: Mouse number. | Treated: Total m.ch. inj. (mg.). | Treated: Interval* (days). | Treated: Gonadotrophic reaction. I. Oe. | Treated: II. Bl.p. | Treated: III. C.l. |
|---|---|---|---|---|---|---|---|---|---|---|---|---|---|
| Subcutaneous injections. | 1 | 1 | 0·2 | 5 | + | − | + | 1 | 2·0 | 5 | − | − | − |
| | | 2 | ,, | ,, | + | − | + | 2 | ,, | ,, | − | − | − |
| | | 3 | ,, | ,, | + | + | + | 3 | ,, | ,, | + | − | + |
| | | 4 | ,, | ,, | + | − | + | 4 | ,, | ,, | + | − | − |
| | | 5 | ,, | ,, | + | − | + | 5 | ,, | ,, | − | − | ± |
| | 2 | 6 | 0·3 | 7 | + | − | + | 6 | 3·0 | 7 | ± | − | ± |
| | | 7 | ,, | ,, | + | − | + | 7 | ,, | ,, | + | − | ± |
| | | 8 | ,, | ,, | + | − | + | 8 | ,, | ,, | − | − | ± |
| | | 9 | ,, | ,, | + | + | + | 9 | ,, | ,, | − | − | − |
| | 3 | 10 | 0·4 | 5 | ± | − | + | 10 | 4·0 | 5 | ± | − | + |
| | | 11 | ,, | ,, | − | − | + | 11 | ,, | ,, | − | − | ± |
| | | 12 | ,, | ,, | + | − | + | 12 | ,, | ,, | − | − | − |
| | | 13 | ,, | ,, | − | + | + | 13 | ,, | ,, | − | − | − |
| | | 14 | ,, | ,, | − | − | ± | 14 | ,, | ,, | ± | − | + |
| Intraperitoneal injections. | 4 | 15 | 0·1 | 1 | + | − | + | 15 | 1·0 | 1 | − | − | ± |
| | | 16 | ,, | ,, | − | − | + | 16 | ,, | ,, | − | − | − |
| | | 17 | ,, | ,, | − | − | − | 17 | ,, | ,, | − | − | ± |
| | 5 | 18 | ,, | 6 | − | − | + | 18 | ,, | 6 | − | − | − |
| | | 19 | ,, | ,, | − | − | − | 19 | ,, | ,, | − | − | − |
| | | 20 | ,, | ,, | ± | − | + | 20 | ,, | ,, | − | − | − |
| | | 21 | ,, | ,, | − | − | + | 21 | ,, | ,, | ± | − | ± |
| | 6 | 22 | 0·2 | 5 | − | − | + | 22 | 2·0 | 5 | − | − | − |
| | | 23 | ,, | ,, | − | − | + | 23 | ,, | ,, | − | − | − |
| | | 24 | ,, | ,, | − | − | + | 24 | ,, | ,, | − | − | − |
| | | 25 | ,, | ,, | − | − | + | 25 | ,, | ,, | − | − | − |
| | | 26 | ,, | ,, | − | − | + | 26 | ,, | ,, | − | − | − |

Abbreviations as in Table I.

### DISCUSSION.

Human chorionic gonadotrophin employed in the experiments contains a follicle-stimulating and luteinizing factor (Prolan A and B of Zondek). The response of immature ovaries to chorionic gonadotrophin consists of follicle growth, followed by bleeding into the follicles and their luteinization. There are, however, indications that chorionic gonadotrophin itself is not a follicle-stimulating factor, and that follicle growth may be the result of stimulation of the test animal's own pituitary, which produces another gonadotrophic hormone synergistic with the injected chorionic gonadotrophin (Selye and Collip, 1933). It is not known whether methylcholanthrene interferes with the production of the synergistic hormone in the hypophysis. No statement, therefore, can be made on the mechanism by which methylcholanthrene inhibits the reaction of the ovaries to gonadotrophin (directly or through the hypophysis). To elucidate this question it would be necessary to employ gonadotrophic hormone of hypo-

TABLE III.—*Examples of the Early Influence of Subcutaneous and Intraperitoneal Injection of 20-methylcholanthrene on Weight Increase in Immature Female Mice (after 8 days).*

| | Strain. | | Treatment on 1st day. | Mean body weight (g.). 1st day. | 9th day. | Percentage increase in weight. |
|---|---|---|---|---|---|---|
| Subcutaneous injections | CBA | Control (8 mice) | 0·2 c.c. olive oil | 8·8 | 12·0 | 36·4 |
| | | Treated (8 mice) | 2·0 mg. m.ch. in 0·2 c.c. olive oil | 8·5 | 11·4 | 34·1 |
| | Strong A | Control (5 mice) | 0·2 c.c. olive oil | 7·9 | 12·0 | 51·9 |
| | | Treated (5 mice) | 2·0 mg. m.ch. in 0·2 c.c. olive oil | 8·3 | 9·3 | 12·0 |
| Intraperitoneal injections. | Strong A | Control (6 mice) | 0·2 c.c. olive oil | 7·3 | 10·8 | 47·9 |
| | | Treated (6 mice) | 2·0 mg. m.ch. in 0·2 c.c. olive oil | 7·5 | 7·1 | – 5·3 |

m.ch. = 20-methylcholanthrene.

physeal origin, as this kind of hormone produces an effect even in animals deprived of the hypophysis.

In the present experiments immature mice were injected subcutaneously or intraperitoneally with a few milligrams of methylcholanthrene from 4–8 days before the gonadotrophic reaction was carried out. A direct absorption of the carcinogen into the ovary following intraperitoneal injection seems likely, and may be responsible for the more pronounced influence on the reaction as compared with the subcutaneous injections. It seems also probable that the absorbed carcinogen acts directly on the ovarian follicles, causing the described deviations in their response. A similar influence on the gonadotrophic reaction has been previously described by Flaks and Ber (1938*a*, *b* and *c*). It was shown in these experiments that immature mice, bearing a transplantable sarcoma, lost their sensitivity to chorionic gonadotrophin. These mice did not develop oestrus, blood points or corpora lutea following the injection of many thousands of mouse units of gonadotrophin. An inhibition of follicle maturation and oestrogen formation was also induced in mice injected with extracts from urine of tumour-bearing men and women (Flaks and Ber, 1939*a*, 1939*b*). It was concluded that the immature ovarian follicles are very sensitive to the influence of toxic products apparently derived from tumour tissue.

The oestrous reaction of the vagina was not always inhibited in the mice treated with methylcholanthrene, even when an inhibition of blood points and luteinization occurred. Therefore, in these mice there was no interference with the production of oestrogen, and the vaginal epithelium did not lose its sensitivity to oestrogen. This result is also in agreement with previous experiments, in which it was shown that in immature mice bearing a transplantable sarcoma the gonadotrophic response of the ovaries was inhibited, while the vaginal response to oestrone remained unaffected (Flaks, Ber and Pakszwer, 1938). The possible production of extra-follicular oestrogen in the theca cells by the follicle-stimulating hormone may to some extent explain the positive vaginal reaction in the carcinogen-treated mice which received gonadotrophic hormone.

Large doses of carcinogenic hydrocarbons exert a general toxic action resulting in a permanent interference with the growth of the body as a whole (Haddow, Scott and Scott, 1937 ; Lees, 1937). Grossly visible changes in lymphoid organs, such as marked atrophy of the spleen, lymph nodes, thymus and bone marrow, have been induced in mice and rats injected with 3:4-benzpyrene (Picard and Laduron, 1934 ; Maisin and Coolen, 1934 ; Picard, 1936). A similar effect on spleen and lymph nodes was observed in the present experiments within a week of intraperitoneal injection of 1·0 mg. or subcutaneous injection of 2·0–4·0 mg. of methylcholanthrene. From a limited number of observations no influence was seen on mitosis in the membrana granulosa of the ovarian follicles. It is not known whether the atrophy of lymphoid tissue has any influence on the response of the immature ovaries to chorionic gonadotrophin. However, the atrophy of the ovaries caused by prolonged treatment with testosterone propionate was shown not to interfere with their normal response to chorionic gonadotrophin (Selye, 1941).

The partial or total inhibition of the formation of corpora lutea may throw some light on the mechanism of abortion produced by carcinogens in pregnant animals. Wolfe and Bryan (1939) showed that daily subcutaneous injection of 5·0 mg. of 1:2:5:6-dibenzanthracene or 3:4-benzpyrene into pregnant rats caused death and resorption of the foetuses. Strong and Hollander (1947) observed a high mortality of embryos of C3H and A mice injected intraperitoneally with 1·0 mg. of methylcholanthrene. These authors suggested that a disturbed vitamin A metabolism, as shown by Goerner and Goerner (1943), may be the cause of the mortality of the embryos. As no symptoms of vitamin A deficiency were observed in the treated females or their offspring, Strong and Hollander assumed that some unknown influence on the corpus luteum may have been the cause of the abortions. The present results show that methylcholanthrene inhibits to a greater or less extent the formation of corpora lutea, and this may be helpful in explaining the mechanism of the abortion following the injection of carcinogenic hydrocarbons. It must be emphasized, however, that the inhibitory influence of methylcholanthrene on the formation of corpora lutea was observed only in immature mice with artificially induced corpora lutea. In adult females injected subcutaneously with 1·0 mg. of methylcholanthrene corpora lutea were found, and such mice became pregnant, and gave birth to normal litters. The influence of intraperitoneal injection of larger doses of methylcholanthrene on corpora lutea has not been investigated, but Lees (1937*a*, 1937*b*, 1937*c*) found no lasting interference with normal oestrous cycles in rats injected with 20·0 mg. of 1:2:5:6-dibenzanthracene.

A growth-inhibitory influence of methylcholanthrene on the comb in chicks was recently described by Hertz and Tullner (1947), who employed testosterone propionate as the growth-promoting agent. A marked inhibition of growth and certain abnormalities of the comb in the carcinogen-treated birds were observed.

The influence of other carcinogenic and non-carcinogenic hydrocarbons on the gonadotrophic reaction of the immature ovaries has not yet been investigated. By this means a relation between the inhibitory influence on the ovary and the carcinogenic properties of the hydrocarbons might be brought to light.

SUMMARY.

(1) 20-Methylcholanthrene was found to exert an inhibitory influence on the action of chorionic gonadotrophin on the ovaries of immature CBA and Strong A mice.

(2) Immature CBA mice injected subcutaneously with 2·0–6·0 mg. or intraperitoneally with 1·0–1·5 mg. of 20-methylcholanthrene, and given chorionic gonadotrophin four to eight days later, showed no blood points and various degrees of inhibition of luteinization. A similar inhibition of luteinization was observed in Strong A mice, but the absence of blood points in these mice could not be considered significant.

(3) The inhibitory influence of 20-methylcholanthrene on follicle haemorrhage and luteinization became apparent in a number of mice before the carcinogen showed its influence on the weight of the body as a whole.

(4) There is no evidence as to whether this inhibition is caused by the direct action of the carcinogen on the ovaries or indirectly through the pituitary gland.

REFERENCES.

Aschheim, S., and Zondek, B.—(1928*a*) *Klin. Wschr.*, **7**, 1404.—(1928*b*) *Ibid*, **7**, 1453.

Elson, L. A., and Haddow, A.—(1947) *Brit. J. Cancer*, **1**, 97.

*Idem* and Warren, F. L.—(1947) *Ibid.*, **1**, 86.

Flaks, J., and Ber, A.—(1938*a*) *Bull. Ass. franç. Cancer*, **27**, 193.—(1938*b*) *Ibid.*, **27**, 239.—(1938*c*) *C. R. Soc. Biol., Paris*, **127**, 1066.—(1939*a*) *Bull. Ass. franç. Cancer*, **28**, 337.—(1939*b*) *C. R. Soc. Biol., Paris*, **130**, 306.

*Iidem* and Pakszwer, R.—(1938) *Ibid.*, **128**, 775.

Goerner, A., and Goerner, M. M.—(1943) *Cancer Res.*, **3**, 833.

Haddow, A.—(1935) *Nature*, **136**, 868.—(1938*a*) *Union Int. contre Cancer*, **3**, 342.—(1938*b*) *J. Path. Bact.*, **47**, 567.—(1938*c*) *Ibid.*, **47**, 581.

*Idem* and Robinson, A. M.—(1937) *Proc. Roy. Soc.*, B, **122**, 442.

*Idem*, Scott, C. M., and Scott, J. D.—(1937) *Ibid.*, **122**, 477.

Hertz, R., and Tullner, W.—(1947) *J. nat. Cancer Inst.*, **8**, 121.

Lees, J. C.—(1937*a*) *Quart. J. exp. Physiol.*, **27**, 161.—(1937*b*) *Ibid.*, **27**, 171.—(1937*c*) *Ibid.*, **27**, 181.

Maisin, J., and Coolen, M. L.—(1934) *C. R. Soc. Biol., Paris*, **117**, 109.

Picard, E.—(1936) *Second Internat. Cancer Congress, Brussels*, Vol. II, p. 120.

*Idem* and Laduron, H.—(1934) *C. R. Soc. Biol., Paris*, **115**, 1739.

Selye, H.—(1941) *J. Endocrinol.*, **2**, 352.

*Idem* and Collip, J. B.—(1933) *Proc. Soc. exp. Biol., N.Y.*, **30**, 647.

Strong, L. C., and Hollander, W. F.—(1947) *J. nat. Cancer Inst.*, **8**, 79.

Wolfe, J. M., and Bryan, W. R.—(1939) *Amer. J. Cancer*, **36**, 359.

Zondek, B.—(1935) "Hormone des Ovariums und des Hypophysenvordenlappens," 2nd ed., Vienna (Springer).

---

ERRATUM.

Dr. Khanolkar reports that the figure 12·7 given for carcinoma of the cervix as percentage of all carcinomas in Indian Christian women in Tables XXIII (p. 192) and XXXIV (p. 208) in the paper "The Racial and Social Incidence of Cancer of the Uterus" (this Journal, Vol. II, pp. 177–212) was an error in his typescript. The correct figure is 30·8. The allusions to the erroneous figure on pp. 193, 210 require deletion.—E. L. K.